# Influence of Herbal Additives on the Physicochemical, Microbiological, Polyphenolic, and Sensory Profile of Green Tea-Based Kombucha

**DOI:** 10.3390/foods14203497

**Published:** 2025-10-14

**Authors:** Magdalena Gantner, Anna Piotrowska, Eliza Kostyra, Ewelina Hallmann, Alicja Ponder, Barbara Sionek, Katarzyna Neffe-Skocińska

**Affiliations:** 1 Department of Functional and Organic Food, Institute of Human Nutrition Sciences, Warsaw University of Life Sciences, Nowoursynowska St. 159c, 02-776 Warsaw, Poland; anna_piotrowska@sggw.edu.pl (A.P.); eliza_kostyra@sggw.edu.pl (E.K.); ewelina_hallmann@sggw.edu.pl (E.H.); alicja_ponder@sggw.edu.pl (A.P.); 2 Bioeconomy Research Institute, Agriculture Academy, Vytautas Magnus University, Donelaicio 58, 44248 Kaunas, Lithuania; 3 Department of Food Gastronomy and Food Hygiene, Institute of Human Nutrition Sciences, Warsaw University of Life Sciences, Nowoursynowska St. 159c, 02-776 Warsaw, Poland; barbara_sionek@sggw.edu.pl (B.S.); katarzyna_neffe_skocinska@sggw.edu.pl (K.N.-S.)

**Keywords:** symbiotic fermentation, functional beverages, plant-based additives, sensory evaluation, QDA, electronic tongue, fermentative microbiota, bioactive compounds, polyphenol profile

## Abstract

Kombucha is a functional beverage with growing popularity due to its health-promoting properties. This study aimed to evaluate the impact of herbal infusions on the quality of green tea-based kombucha. Four variants were prepared: a control (K1) and three experimental samples combining 70% green tea with 30% (*v*/*v*) *Mentha spicata* (K2), *Hibiscus sabdariffa* (K3), or *Clitoria ternatea* (K4). Fermentation lasted four days at 24 ± 1 °C. Physicochemical parameters, polyphenol profile (HPLC), microbiological safety, and sensory quality were assessed using QDA and electronic tongue analysis. K3 showed the highest polyphenol content (291 mg/L), especially catechins. K4 achieved the highest overall sensory quality due to its fruity aroma, balanced sweet-sour taste, and favorable microbiological profile. K2 had the lowest caffeine content (114 mg/L) and a distinct minty flavor. All samples were microbiologically safe. Herbal additives influenced fermentative microbiota: K3 had fewer acetic acid bacteria, while K4 had the highest lactic acid bacteria count. Electronic tongue analysis confirmed sensory panel results and revealed distinct taste profiles among the variants. Herbal infusions significantly enhance the nutritional and sensory properties of kombucha. Their use offers a promising strategy for developing functional beverages with tailored characteristics.

## 1. Introduction

The increasing prevalence of diet-related diseases such as obesity, type 2 diabetes, and cardiovascular disorders has heightened global interest in functional foods-products that offer health benefits beyond basic nutritional value. Functional foods are typically enriched with bioactive compounds such as vitamins, minerals, polyphenols, dietary fiber, and beneficial microorganisms, including probiotics, which contribute to improved physiological and metabolic functions [1]. Among the most dynamic segments of this category are functional beverages, which are gaining popularity due to their convenience, palatability, and potential health-promoting properties [2].

Kombucha, a fermented tea beverage originating from Asia, has become a leading product in the functional beverage market. It is produced by fermenting sweetened tea with a Symbiotic Culture of Bacteria and Yeast (SCOBY), resulting in a slightly acidic, effervescent drink rich in organic acids, polyphenols, vitamins, and live microbial cultures [3,4]. Kombucha has been associated with a wide range of health benefits, including antioxidant, antimicrobial, anti-inflammatory, hepatoprotective, and blood glucose-regulating effects [5,6,7].

Despite the growing popularity of kombucha, there are still many gaps in scientific knowledge about it. Much research focuses on chemical composition without directly linking it to biological activity, and there is a lack of standard protocols for fermentation time, microorganism characterization, and functional validation [8]. Furthermore, the physiological significance of bioactive compounds such as polyphenols is often assumed rather than demonstrated, and the potential probiotic functionality of microorganism populations such as lactic acid bacteria (LAB) is rarely studied in depth [9,10,11].

Recent studies have shown that the chemical composition, biological activity, and sensory properties of kombucha are strongly influenced by the type of tea used, fermentation conditions, and plant-based additives such as fruits or herbal extracts [11,12]. Andrade et al. [13] emphasize that the diversity of raw materials—including black, green, and oolong teas, as well as herbs—affects the content of bioactive compounds such as polyphenols, flavonoids, and organic acids, which are responsible for the beverage’s antioxidant, antimicrobial, and detoxifying properties. For example, the use of pineapple-peel infusions resulted in increased polyphenol content and higher antioxidant activity compared to traditional formulations [14]. Additionally, the profile of volatile aromatic compounds—including esters, phenols, aldehydes, and organic acids—plays a significant role in shaping the sensory characteristics of kombucha [15].

Herbal additives in particular offer promising opportunities to increase bioactivity and consumer appeal. However, few studies have systematically evaluated the effect of individual herbs on fermentation dynamics, microbiological profile, and sensory properties.

This study fills these gaps by analyzing the effect of three herbal infusions-*Mentha spicata, Hibiscus sabdariffa*, and *Clitoria ternatea*—on the physicochemical, microbiological, polyphenolic, and sensory properties of green tea-based kombucha. *Mentha spicata* is known for its minty aroma and anti-inflammatory properties [16,17]; *Hibiscus sabdariffa* is rich in anthocyanins and organic acids with antioxidant and cardioprotective potential [18,19]; and *Clitoria ternatea* contains flavonoids and anthocyanins associated with neuroprotective and antioxidant effects [20]. By comparing the control sample with three experimental variants, this study aims to determine how herbal enrichment modulates fermentation outcomes and contributes to the development of functional beverages with enhanced health and sensory properties.

## 2. Materials and Methods

### 2.1. Materials

This study used loose green tea (*Camellia sinensis*) of the Gunpowder variety, sourced from Ahmad Tea (United Kingdom), with the raw material originating from China. Herbs were sourced from the Polish Dary Natury company: spearmint (*Mentha spicata*), dried hibiscus petals (*Hibiscus sabdariffa*), and dried butterfly pea herb (*Clitoria ternatea*). All raw materials came from a single production batch and were stored in a dry, cool place until use. Sucrose and kombucha starter cultures, SCOBY, were also used, occurring in the form of acidic broth and a cellulose layer (SCOBY floats on the surface of the liquid). The starter cultures used in this study were stored in a refrigerator (4 °C).

### 2.2. Preparation of Kombucha–Type Beverages

Green tea (0.8% *m*/*v*) was brewed in 1 L of water at 80 °C for 5 min in sterile flasks. In this study, a green tea infusion was used at a concentration of 0.8% *m*/*v*, which corresponds to 8 g of dried leaves per 1 L of water. This is a higher value than typically used in home settings (2–3 g/L), but it aligns with research practices. Both Zou et al. and Adrade [8,13] indicate that tea concentrations ranging from 5 to even 10 g/L are commonly used in kombucha research, depending on the experimental objective. The use of a higher concentration aimed to enhance the extraction of bioactive compounds such as polyphenols and caffeine, which was reflected in the chemical and sensory results. Herbal infusions (spearmint, hibiscus, butterfly pea) were prepared separately in the same proportion (1% *m*/*v*) by pouring boiling water (100 °C) over the herbs and steeping for 10 min. The control sample (K1) contained only green tea infusion, while variants K2–K4 included a 30% (*v*/*v*) addition of spearmint, hibiscus, and *Clitoria ternatea* infusions, respectively. After brewing, all infusions were filtered through nylon membrane filters (0.45 μm, diameter 25 mm, Sigma-Aldrich, Poznań, Poland) into sterile glass jars. Sucrose was then added at a concentration of 8% (*m*/*v*) and stirred until fully dissolved. After cooling to room temperature, the infusions were filtered again and inoculated with 10% volume of previously fermented kombucha and a SCOBY biofilm. The SCOBY culture used in this study was obtained from a commercial Polish supplier and consisted of a cellulose biofilm and acidic fermentation broth from a previous kombucha batch. The inoculum (10% *v*/*v*) had a pH of approximately 3.2 and an extract content of approximately 6.50% Brix. Although the microbial composition of the SCOBY was not characterized in this study, it is expected to include acetic acid bacteria (e.g., *Acetobacter* spp.), lactic acid bacteria (e.g., *Lactobacillus* spp.), and yeasts (e.g., *Saccharomyces* spp.), consistent with previous reports [3,20]. Fermentation was carried out under aerobic conditions (containers covered with sterile gauze) at 24 ± 1 °C for 4 days, in a dark room with a relative humidity of 60–70%. The fermentation time was selected based on previous studies that yielded optimal sensory and microbiological properties of the beverage [16]. After fermentation, the beverages were filtered and stored at 4 °C until analysis. The fermentation period of four days was selected based on previous studies, indicating that a duration of 4–7 days ensures optimal antioxidant activity and favorable sensory properties of the beverage [21].

All samples were prepared from a single fermentation batch per variant. Technical replicates (n = 3) were used for analytical procedures, although biological replicates were not included.

### 2.3. pH Measurement

A total of 15 mL of each sample was transferred into 25 mL beakers. The pH was measured using a calibrated Hanna Instruments HI2211 pH meter by immersing the electrode in the solution for a few seconds. Each measurement was performed in triplicate.

### 2.4. Determination of Extract Content (Brix)

The extract content was determined using an ATAGO PAL-1 optical refractometer on the Brix scale. A few drops of the beverage were placed on the prism, covered with a transparent lid, and the result was read in daylight. Each sample was analyzed in triplicate.

### 2.5. Determination of Polyphenol and Caffeine Contents

The content of polyphenolic compounds as well as caffeine compounds was determined using high-performance liquid chromatography (HPLC) at the Department of Organic Food at the University of Life Sciences (SGGW), following the procedure by Górecki et al. [22]. Kombucha samples (2 mL) were mixed with 3 mL of 80% methanol in plastic tubes, incubated in an ultrasonic bath (10 min, 30 °C), and then centrifuged (6000 rpm, 10 min, 0 °C). The supernatant was transferred to HPLC vials. The analysis was performed using a Phenomenex Fusion 80-A RP column (250 × 4.6 mm), with a mobile phase of acetonitrile/deionized water acidified with H_3_PO_4_ (pH 3.0), flow rate of 1 mL/min, and UV-Vis detection in the range of 280 (for caffeine and phenolic acids) and 340 nm (for flavonoids). Standards (purity ≥99%) were obtained from Fluka and Sigma-Aldrich. The group of phenolic acid standards included the following: gallic acid, chlorogenic acid, caffeic acid, p-coumaric acid, ferulic acid, vanillic acid, t-cinnamic acid. The group of flavonoid standards included the following: epigallocatechin, catechin, quercetin-3-O–rutinoside, naringin, kaempferol-3-O-glucoside, quercetin, kaempferol, and caffeine. Each sample was analyzed in four replications.

### 2.6. Sensory Evaluation

The sensory characteristics of the kombucha samples were evaluated using the Quantitative Descriptive Analysis (QDA) method following the guidelines of PN-EN ISO 13299:2016 [23]. Twenty-two attributes were selected and defined according to the profiling procedure—clarity (visual assessment), nine odor attributes (acetic, sharp, sour, fermentative, sweet, fruit, tea, mint, herbal), ten flavor/taste cues (vinegar, sharp, tingling, sour, sweet, fruit, tea, mint, herbal, pungency), and overall sensory quality defined as the impression of the harmony of all examined attributes, with no or only a slight intensity of negative cues. The intensity of attributes was measured on a linear unstructured scale ranging from 0 (imperceptible) to 10 cm (very intense). Regarding overall sensory quality, anchors on the scale edges were from low to high. The evaluation took place in the Laboratory of Sensory Analysis at the Institute of Human Nutrition Sciences, Warsaw University of Life Sciences. The laboratory meets the requirements for sensory laboratories specified in the PN-EN ISO 8589:2010/A1:2014-07 [24] standard. It is equipped with the system ANALSENS. The evaluation was conducted by a specially trained sensory panel consisting of ten experts who were qualified according to PN-EN ISO 8586:2014-03 [25]. The samples were analyzed in two independent sessions, and the presented results are the average of 20 evaluations (10 assessors × 2 sessions). Each assessor received 20 mL of the kombucha sample in covered plastic beakers (150 mL). The samples were coded individually and presented in a sequential monadic test. Still water was provided as a factor to neutralize the taste between evaluations of subsequent samples.

### 2.7. Analysis of Chemosensors

The assessment of the kombucha samples’ basic tastes was carried out using an A stree electronic tongue system (Alpha MOS, Toulouse, France), which identifies taste-relevant organic and inorganic substances dissolved in liquids. The e-tongue consists of an automatic sampler, a reference electrode of Ag/AgCl, seven potentiometric chemical sensors (sensor set #7: AHS, SCS, ANS, CPS, NMS, CTS, PKS), as well as a data acquisition system. Taste detection is based on potentiometric analysis, where the electrical potential difference between each sensor and the Ag/AgCl reference electrode at equilibrium is recorded as the response signal. Before measurement, the sensors were conditioned and calibrated using a 0.01 mol/L hydrochloric acid (HCl) solution. This same solution, along with monosodium glutamate (MSG) and sodium chloride (NaCl), was used to verify the sensitivity and accuracy of the sensors. The sourness, saltiness, and umami were measured using 0.1 M HCl, 0.1 M NaCl, and 0.1 M MSG as reference materials for the electronic tongue sensor, respectively. To determine sourness, saltiness, and umami of the samples, solutions of 0.1 M HCl, 0.1 M NaCl, and 0.1 M MSG were utilized as reference materials for the electronic tongue sensors. Each kombucha sample was measured ten times, and data from three stabilized points were selected for further analysis. Sensor signals were recorded at one-second intervals over a 120-s period to ensure sufficient data acquisition. Following each measurement cycle, the sensors were rinsed in ultrapure water for 10 s to stabilize before the next sample evaluation. Individual sensors targeted specific taste modalities: AHS (sour), CTS (salty), NMS (umami), ANS (sweet), SCS (bitter), while PKS and CPS served broader analytical functions.

### 2.8. Microbiological Analysis

Microbiological analyses were performed on dedicated culture media (Biokar diagnostics, France) for each of the tested microorganism groups in accordance with PN-EN ISO 7218:2008/A1:2013-10; PN-ISO 15214:2002; PN-ISO 21527-1:2009; PN-EN ISO 7932:2005; PN-EN ISO 21528-2:2017-08 [26,27,28,29,30]. Lactic acid bacteria (LAB) were counted by plate plating on MRS medium (Merc, Germany), total viable count (TVC) was counted on nutrient agar (LabM, Heywood, UK), *Enterobacteriaceae* (ENT) were counted on VRBG agar (LabM, Heywood, UK), and *Bacillus cereus* was counted on PEMBA agar (LabM, Heywood, UK). The plates were incubated at 37 °C for 48 h. Acetic acid bacteria (AAB) were counted using a modified GCA (Glucose Calcium Carbonate and Agar) solid medium according to Neffe-Skocińska et al. [22]; incubation was carried out at 28 °C for 72 h. Yeast and mold counts were determined on YGC agar (Biomaxima, Lublin, Poland), incubated at 25 °C for 120 h. Results were expressed as the logarithm of colony-forming units per mL of product (log CFU/mL). Microbial analyses were performed in three replicates for each of the study’s kombucha variants. The results were analyzed using the criteria contained in Commission Regulation (EC) No. 1441/2007 of 5 December 2007 amending Regulation (EC) No. 2073/2005 on microbiological criteria for foodstuffs. Results are expressed in colony-forming units per milliliter of product (CFU/mL).

### 2.9. Statistical Analysis

Data were subjected to one-way analysis of variance (ANOVA) with Tukey’s post hoc test (*p* ≤ 0.05). Prior to analysis, the normality of distribution was verified using the Shapiro-Wilk test. Homogeneity of variances was not formally tested (e.g., Levene’s test), which is acknowledged as a limitation of the current statistical approach.

Results were presented as means ± standard deviation. Homogeneous groups were marked with the same letters. Analyses were performed using Microsoft Excel 365 (Microsoft Corp., Redmond, WA, USA) and Statistica 13.3 (StatSoft Inc., Kraków, Poland). Principal Component Analysis (PCA) of the e-tongue data from examined samples was conducted using AlphaSoft software (Alpha MOS S.A., Toulouse, France).To illustrate the similarities and differences between bioactive compounds and sensory characteristics of kombucha samples, a Principal Components Analysis (PCA) was conducted using XLSTATS version 2021 software, developed by Addinsoft (Paris, France).

## 3. Results and Discussion

As a result of the conducted study, significant differences were observed in the physicochemical, sensory, and microbiological characteristics of kombucha beverages prepared with various herbal infusions. The findings provide valuable insights into how the addition of mint K2, hibiscus K3, and *clitoria* K4 influences fermentation dynamics, bioactive compound profiles, and consumer-relevant quality attributes. These results are consistent with previous research on functional beverages and highlight the potential of herbal kombucha as a source of health-promoting compounds and sensory diversity [8,13].

### 3.1. Physicochemical Analysis

#### 3.1.1. Changes in pH During Kombucha Fermentation

**Figure 1:**
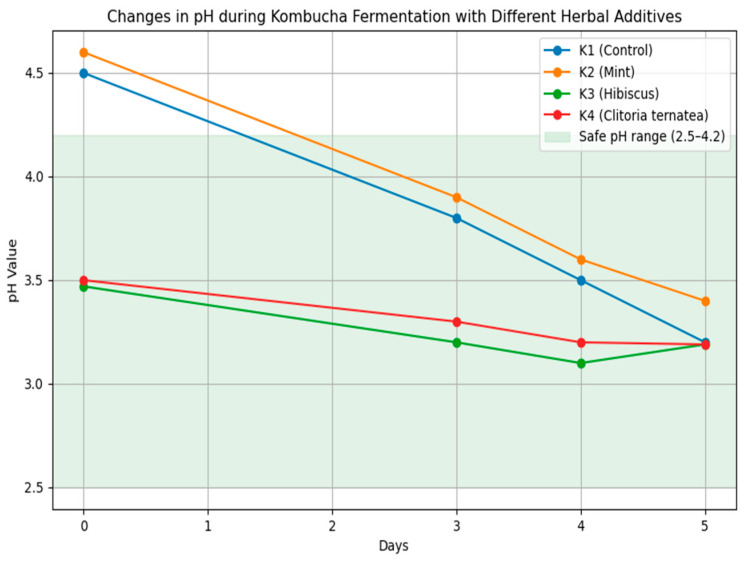
Changes in pH during kombucha fermentation in variants with different herbal additives. The green area indicates the safe pH range (2.5–4.2). Each line represents a different kombucha variant: K1 (green tea control), K2 (mint), K3 (hibiscus), and K4 (*clitoria*).

The pH value is a key indicator of the progress of kombucha fermentation and its safety for consumption [31,32]. In this study, pH was monitored on days 0, 3, 4, and 5 (Table 1; Figure 1). The initial pH values differed significantly due to the acidity of the herbal infusions. The hibiscus variant (K3) had the lowest initial pH (3.47), which is consistent with its high content of organic acids, such as ascorbic and malic acid [31,32]. Galus and Podolska [18] determined that at a concentration of 2.5% hibiscus flowers, the pH of the infusion is 2.58. During fermentation, the pH decreased as a result of the production of organic acids, mainly acetic acid, which is responsible for the acidity of kombucha [18]. Sample K1 (control) ultimately had a pH of 3.21, which is almost identical to the 2022 study by Costa et al. [32] in which green tea kombucha had a pH of 3.2. The largest decrease in pH was observed between days 0 and 3. By day 5, all samples had reached pH values within the safe range (2.5–4.2) [32], with the mint variant (K2) having the highest final pH (3.40) and the variants with hibiscus (K3) and *clitoria* (K4) having the lowest (3.19), which is consistent with previous studies [33]. This trend in pH reduction is consistent with previous studies by Costa et al. [32] and Galus & Podolska [18], who observed similar dynamics of acidification during kombucha fermentation, particularly in formulations enriched with hibiscus.

#### 3.1.2. Extract Content

The extract content, expressed as a percentage of Brix, reflects the concentration of soluble substances, mainly residual sugars, in kombucha samples after fermentation. As shown in Table 2, statistically significant differences (*p* ≤ 0.05) were observed between the four variants. The control sample (K1), prepared exclusively from green tea infusion, showed the lowest extract content (6.50 ± 0.16%), indicating the highest degree of sugar utilization by microorganisms during fermentation; in contrast, the highest extract content was found in samples K3 (hibiscus) and K4 (*clitoria*), which reached a value of 7.83 ± 0.05%, suggesting lower sugar consumption.

These results are consistent with previous studies by Majid et al. [33], who reported a similar extract level (6.35%) in green tea kombucha fermented under similar conditions (22 °C, 7% sucrose, and 7 days). The lower extract content in sample K1 may result from more efficient metabolism of microorganisms, probably due to the absence of phenolic compounds or organic acids, which are present in herbal infusions and may inhibit microbial activity. It was noted that the addition of hibiscus and *clitoria* infusions could affect the activity of microorganisms, which may be related to the presence of bioactive compounds. Hibiscus, rich in organic acids such as ascorbic and malic acids [18], may have altered the fermentation environment, while *clitoria* contains anthocyanins and flavonoids, which may affect the dynamics of the microflora [34]. These compounds may have exhibited mild antimicrobial activity or altered the osmotic balance, limiting the rate of sugar metabolism. The sample with mint (K2) showed an indirect extract content (7.00 ± 0.22%), which may indicate a moderate effect of the compounds contained in mint on fermentation. Similar observations were made by Pawluś and Kolniak-Ostek [35], who noted that kombucha with mint showed a slower rate of sugar degradation compared to traditional variants. Bartolomedii et al. [5] also noted that a higher extract content after fermentation may indicate lower sugar consumption (e.g., due to the action of antimicrobial compounds in the substrate), shorter fermentation time, or lower microorganism activity. In turn, a lower extract content means that microorganisms have effectively converted sugars into fermentation products, which may be beneficial from a health perspective (lower residual sugar content).

The results obtained indicate that the type of herbal infusion used significantly affects the metabolic activity of SCOBY, which is most likely due to differences in the chemical composition of the infusions. This emphasizes the importance of substrate selection in the design of kombucha recipes as it directly affects the fermentation process and, consequently, the nutritional value and sensory profile of the finished drink.

#### 3.1.3. Polyphenol and Caffeine Content

**Figure 2:**
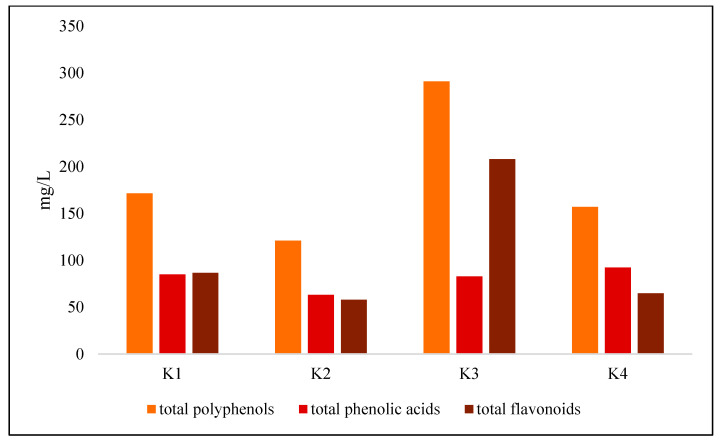
Individual phenolic groups in kombucha samples. Values given as mean (n = 3). K1c—ontrol, K2—30% mint, K3—30% hibiscus, K4—30% *clitoria*.

The content of phenolic groups in the tested samples is presented in Figure 2. The total polyphenol content in the tested kombucha samples differed significantly depending on the herbal additive used. The highest concentration was found in the sample with hibiscus (K3), which amounted to 291.00 ± 3.95 mg/L and was significantly higher than in the control sample (K1) based solely on green tea (171.57 ± 2.72 mg/L). Hibiscus, as a raw material rich in flavonoids, especially catechins, has strong antioxidant potential, which has been confirmed by previous studies on its use in kombucha fermentation [35]. These findings are consistent with the results of Andradae et al. [13], who demonstrated that herbal additives such as hibiscus significantly increase the concentration of bioactive compounds in kombucha, including polyphenols and flavonoids. The high polyphenol content in variant K3 may suggest antioxidant potential, although its physiological significance depends on bioavailability and biological activity, which were not the subject of this study.

Kombucha with *Clitoria ternatea* (K4) contained an intermediate amount of polyphenols (157.17 ± 3.07 mg/L), significantly more than K2 but less than K3. These results partially confirm the hypothesis that herbal additives increase the polyphenol content, with hibiscus proving to be particularly effective. Nevertheless, as demonstrated in the literature [1,2,3], polyphenol content does not always correlate with their biological activity, which highlights the need for tests such as DPPH, ABTS, or FRAP.

It is worth noting that fermentation can increase the bioavailability of polyphenols by breaking down complex phenolic compounds into more biologically active compounds [36]. Furthermore, the presence of tea sediment in the beverage may further increase the content of phenolic compounds, as demonstrated in studies on kombucha produced from Vine Tea and Sweet Tea [37].

Among the phenolic acids, the highest concentration of caffeic acid was recorded in the control sample (39.81 ± 0.44 mg/L), followed by samples K4 (29.26 ± 0.72 mg/L) and K3 (27.15 ± 0.35 mg/L) (Table 3). In contrast, p-coumaric acid was characteristic of the samples with hibiscus and butterfly pea (28.95 ± 0.35 and 29.90 ± 0.90 mg/L, respectively), indicating their richness in this compound. The control sample contained only 3.05 ± 0.07 mg/L, highlighting the impact of herbal additives on the phenolic profile.

In the flavonoid, catechin was the predominant flavonoid, especially in sample K3 (165.82 ± 0.51 mg/L), accounting for over 57% of all polyphenols in this sample. This result supports previous findings on the high catechin content in hibiscus and their role in enhancing the antioxidant activity of kombucha [38]. Other important flavonoids included quercetin-3-O-rutinoside (with the highest levels in K1 and K4) and quercetin, which reached its highest concentration in the mint sample (11.16 ± 0.75 mg/L), reflecting the specific phytochemical composition of this plant [35].

##### Caffeine Content

The caffeine content varied significantly between samples (Table 3). The highest concentration was found in the control sample (K1)—321.91 ± 3.50 mg/L. It was significantly higher than in samples with herbal additives. This result is consistent with expectations as green tea was the only source of caffeine, and its content was reduced by 30% in herbal variants. The lowest caffeine concentration was found in the sample with mint (K2)—114.34 ± 5.47 mg/L, which can be explained by the absence of caffeine in mint and the possible degradation of caffeine during fermentation [39]. Samples K3 and K4 showed intermediate values (162.60 ± 1.02 and 172.08 ± 1.18 mg/L, respectively), with no significant differences between them. It is worth noting that the caffeine content in the control sample was almost twice the average content of this alkaloid in green tea infusion (approx. 167 mg/L) [40], which may suggest increased extraction or concentration of caffeine as a result of fermentation [41,42].

Herbal additives significantly affected the polyphenol and caffeine profile of kombucha drinks. Hibiscus significantly increases the polyphenol content, especially flavonoids, while mint and *Clitoria ternatea* contribute characteristic phenolic acids and flavonoids. At the same time, herbal additives reduce the caffeine content, which may be beneficial for consumers looking for functional beverages with low caffeine content and high antioxidant potential.

### 3.2. Microbiological Analysis

The lowest count of acetic acid bacteria (AAB) was recorded in the variant with hibiscus (K3—*Hibiscus sabdariffa*), which may indicate an inhibitory effect of organic acids present in hibiscus, such as ascorbic and malic acids. The variants with spearmint (K2—*Mentha spicata*) and butterfly pea (K4—*Clitoria ternatea*) showed intermediate values (2.26 and 2.39 log CFU/mL, respectively), with no significant differences compared to the control (Table 4).

The count of lactic acid bacteria (LAB) in all tested kombucha variants was low (below 4 log CFU/mL). This observation aligns with previous studies describing the microbial diversity of SCOBY cultures, which can vary depending on the substrate used [3]. A low LAB count is typical for kombucha, and results from the acidic fermentation environment (pH < 4.0) [43]. It should be emphasized that although variant K4 showed the highest number of lactic acid bacteria, its probiotic properties cannot be clearly determined without identifying the strains and assessing their survival rate.

The yeast and mold counts (YMCs) ranged from 6.09 to 6.32 log CFU/mL across all samples, with the highest value in K4. The dominance of yeasts over bacteria is characteristic of the SCOBY microbiome, and reflects the symbiotic nature of the fermentation process [44].

There were significantly higher total viable counts (TVCs) in samples K1 and K2 (*Camellia sinensis* and *Mentha spicata*) compared to K3 and K4 (*Hibiscus sabdariffa* and *Clitoria ternatea*); this may suggest that the herbal additives in K3 and K4 exhibited mild antimicrobial activity, likely associated with the presence of polyphenols and flavonoids [5,35].

Importantly, no *Enterobacteriaceae* or *Bacillus cereus* bacteria were detected in any of the samples, which is essential for the safety and quality of the beverages. The *Enterobacteriaceae* family includes bacteria that cause food poisoning, such as *Salmonella* spp., *Yersinia enterocolitica*, *Escherichia coli*, *Shigella* spp., and *Cronobacter* spp. [9,10]. During the fermentation of kombucha, compounds with antimicrobial properties are produced, e.g., organic acids, ethanol, and polyphenols [43,45]. Fabricio et al. [46] also did not detect the presence of the *Enterobacteriaceae* family after 7 days of fermentation of sweetened green tea at a controlled temperature (from 28 to 25 °C). The results highlight the influence of herbal additives on the fermentative microbiota of kombucha and suggest their potential use in modulating the fermentation process and enhancing product safety.

### 3.3. Sensory Evaluation and Analysis of Chemosensors

Table 5 presents the mean results of the kombucha samples’ sensory evaluation along with their statistical interpretation. The data indicate that the addition of herbal infusions significantly influenced the beverages’ sensory profile and overall sensory quality. Clarity of the kombucha samples with added herbal ingredients, *Mentha spicata* (K2, *Hibiscus sabdariffa* (K3), and *Clitoria ternatea* (K4), was significantly higher compared to the control sample. These differences may be attributed to the presence of clarifying and antioxidant compounds such as anthocyanins and flavonoids [12]. Concerning the odor profile, the K2 sample (Mentha spicata) was characterized by the significantly lowest intensity of acetic and fermentative odor; this could be connected with the presence of mint additives, which could influence the volatile compound profile and mask typical odors associated with the production process. Samples K3 and K4 were characterized by a slightly higher intensity of fruit odor, although these differences were not statistically significant. The examined samples had similar intensity of the sharp, sour, tea, and sweet odors; however, it can be noticed that the slightly higher intensity of sharp and sour odors and the lower intensity of sweet odor were found in the control sample.

The kombucha beverages differed significantly in the taste/flavor profiles. The sample with *Mentha spicata* (K2) profile was dominated by a mint flavor, while kombucha with *Hibiscus sabdariffa* (K3) and with *Clitoria ternatea* (K4) had significantly higher intensity of the fruit note in comparison to K1 and K2 samples. The sour and sharp attributes were most pronounced in samples K1 and K2. The control sample was also more tingling and pungent. The intensity of the tea and herbal flavors remained similar across the samples.

Differences in the sensory profile of the kombucha beverages influenced their overall sensory quality, defined as the appropriate balance of key sensory attributes and absence or only slight intensity of negative notes. The sensory quality of the beverages with herbal additives was statistically significantly higher compared to the control sample. Among the beverages evaluated, sample K4 was characterized by the significantly lowest intensity of acetic, sharp, tingling cues, and sour taste, resulting in the highest overall sensory quality.

**Figure 3:**
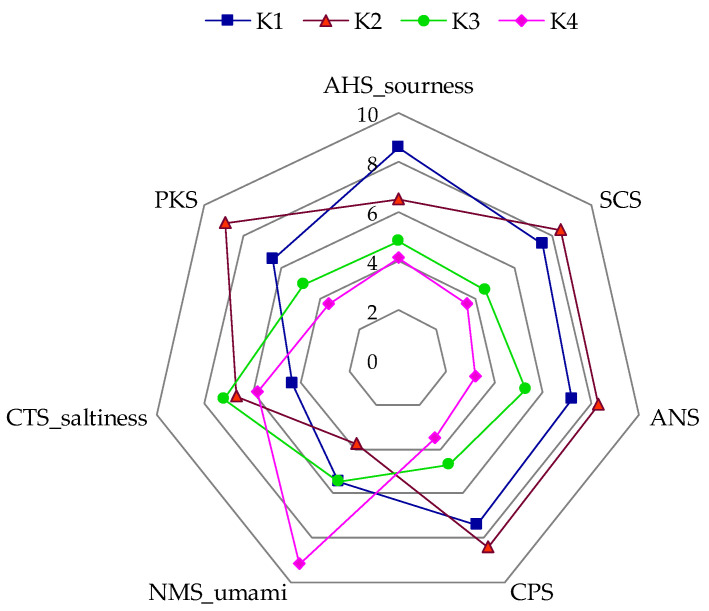
Radar map of e-tongue data for the kombucha samples—e-tongue analysis. K1—control, K2—30% mint, K3—30% hibiscus, K4—30% *clitoria*. Each line represents the sensor response for a specific kombucha variant.

In addition to the sensory panel assessment, an analysis of the chemosensors was conducted using an e-tongue. The radar chart (Figure 3) illustrates the comparative taste profiles of four kombucha samples (K1–K4) across basic tastes measured by potentiometric sensors. The results revealed differences in the intensity of basic tastes among the kombucha beverages. The control sample (K1) showed the highest intensity in AHS (sourness) and high-intensity SCS (bitterness) sensors, suggesting a more acidic and bitter profile typical of unflavored green tea kombucha. It was found that kombucha with *Clitoria ternatea* (K4) stood out with higher values in NMS (umami). The response of the AHS (sourness) and SCS (bitterness) sensors was similar in samples K3 and K4.

When comparing the results obtained using the electronic tongue with the sensory panel assessments, it can be concluded that there is a high correlation in the assessment of sour taste (r = 0.94). However, for the sweet taste, the correlation coefficient is negative (r = −0.62). It is noteworthy that in the sensory panel assessment, the intensity of the sweet taste was quite high in the K4 sample, while in the case of the e-tongue, the response of the ANS sensor responsible for sweet taste was the lowest for this sample. However, it should be noted that sample K4 was characterized by the highest response value of the NMS sensor (umami). Umami not only shapes the palatability of various products but also plays a significant role in enhancing the perception of sweetness through sensory and neurophysiological mechanisms [47]. This underscores the complex nature of taste perception and the interplay of different taste components. Although the scientific literature does not explicitly confirm the presence of glutamic acid in *Clitoria ternatea*, the occurrence of amino acids in this plant—particularly in the context of its classification within the *Fabaceae* family—suggests a high likelihood of its presence [47,48]. This may be important for the perception of umami taste, especially in infusions or fermented products containing *clitoria*. Sweet taste perception is a complex process involving not only the activation of taste receptors but also the integration of olfactory cues. Orthonasal olfaction (smelling through the nose) and retronasal olfaction (aroma perceived from the mouth during eating) significantly enhance sweetness perception. Volatile compounds associated with sweet aromas can amplify perceived sweetness [49]. Samples K3 and K4 were characterized by a higher intensity of the fruity note in the sensory profile, which could have influenced the panellists’ perception of sweet taste and influenced differences between sensor evaluation and chemosensory analysis.

**Figure 4:**
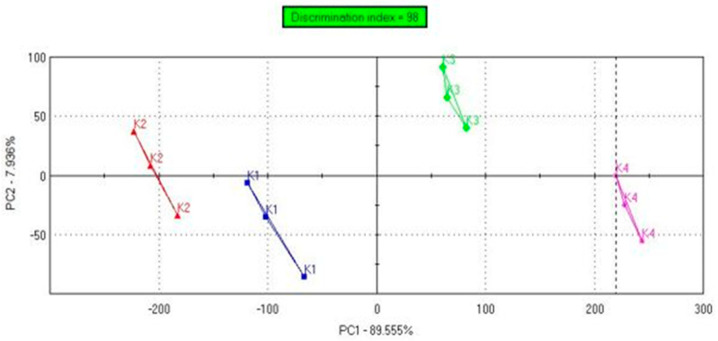
PCA plot of the similarities and differences in the kombucha samples’ taste—e-tongue analysis. Each line represents the PCA distribution of sensor data for a specific kombucha variant.

The PCA plot (Figure 4) visualizes the distribution of four kombucha samples (K1–K4) based on their taste-related sensor data. The discrimination index of 98 confirms good separation between the tested beverages. The variance contribution rate of PC1 was 89.55%, and PC2 was 7.93%. The resulting PCA image indicates that each herbal additive—*Mentha spicata* (K2), *Hibiscus sabdariffa* (K3), and *Clitoria ternatea* (K4)—modified the taste profile of the green tea kombucha (K1). Sample K4 was the most different from the control sample regarding basic tastes.

The results of both the sensory and chemosensory studies clearly indicate that the addition of herbal additives significantly affects the sensory characteristics of kombucha. Regardless of the type of herbal additive used (*Mentha spicata, Hibiscus sabdariffa*, or *Clitoria ternatea*), a significant improvement in overall sensory quality was observed compared to the control sample. The *Clitoria ternatea* variant (K4) achieved the highest score, making it the most promising formulation. Our findings support the use of botanical additives in the development of functional beverages that offer enhanced sensory quality and health benefits. Future studies should include consumer research to evaluate the acceptance of the kombucha beverages we developed.

As reported by Shahbazi et al. [50], different herbs introduce distinct flavor and aroma notes that can enhance consumer acceptance. Neffe-Skocińska et al. [51] also emphasized the role of fermentation conditions, such as temperature and time, in shaping sensory quality. Their findings indicated that fermentation at 25 °C yielded the most favorable sensory outcomes. Dartora et al. [52] highlighted the interaction between SCOBY microorganisms and bioactive compounds in herbal infusions, which may modulate enzymatic activity and, consequently, the sensory profile of kombucha. Ivanišová et al. [53] showed that kombucha made from black tea had a pleasant fruity-sour taste, which was rated as the most acceptable by consumers.

**Figure 5:**
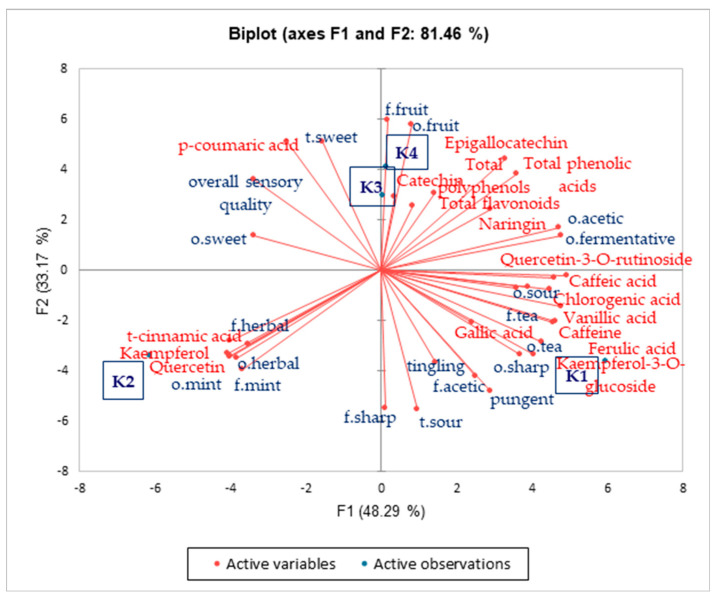
PCA plot of similarities and differences between bioactive compounds and sensory characteristics of kombucha samples (K1—control, K2—30% mint, K3—30% hibiscus, K4—30% *clitoria*). Lines indicate the clustering of sensory attributes and bioactive compounds associated with each kombucha variant.

A PCA was conducted to examine the relationship between bioactive compounds and sensory profiles (Figure 5). It can be observed that, near sample K1, compounds such as kaempferol-3-O-glucoside, caffeine, ferulic acid, gallic acid, vanillic acid, chlorogenic acid, as well as sensory attributes like sour odor, tea odor and flavor, sharp odor, pungency, tingling, and acetic flavor clustered together. Compared to K1, different sensory characteristics were represented by sample K2, where the sensory profile was defined by herbal odor and flavor, mint odor and flavor, and the presence of t-cinnamic acid, kaempferol, and quercetin compounds. Samples K3 and K4 showed similar sensory attributes with fruit odor and flavor and sweet taste, featuring notable compounds such as epigallocatechin, catechin, and p-coumaric acid. These samples were more distant compared to K1 and K2. In the literature, it is emphasized that phenolic compounds contribute to food quality from a sensory perspective by enhancing color, aroma, taste, and flavor [53,54]; however, polyphenolic compounds are known to cause bitterness and astringency in various beverages (e.g., tea) [55]. In our study, bioactive substances likely contributed to the sensory characteristics of the samples (e.g., the intensity of tea odor and flavor, pungency, and tingling). However, establishing a direct relationship between bioactive compounds and sensory traits is challenging because the interactions among key ingredients can also influence taste and flavor properties of products [56].

### 3.4. Limitations of This Study

Despite the valuable findings obtained, several limitations should be considered when interpreting the data. Firstly, only three types of herbal additives were used, which may restrict the generalizability of the results to other plant-based ingredients. Secondly, the fermentation period was limited to four days, which—although consistent with previous assumptions—may not fully reflect long-term microbiological and biochemical changes. In future studies, it would be worthwhile to consider different fermentation periods (e.g., 7 or 14 days) to determine the optimal time for different quality parameters. Furthermore, this study was conducted in vitro, without in vivo experiments that could confirm the potential health-promoting effects of the beverages. Additionally, product stability during storage was not assessed, which may be relevant for evaluating shelf life and safety in the context of commercial applications. Despite a detailed analysis of the polyphenol and flavonoid profile, this study did not include direct biological activity tests, such as antioxidant capacity assays (e.g., DPPH, ABTS, and FRAP) and antimicrobial activity. The content of bioactive compounds does not always translate into their functionality, which depends, among other things, on chemical structure, bioavailability, and interaction with other ingredients. In future studies, we plan to perform functional tests to confirm the health-promoting properties of beverages. Nevertheless, this study provides a meaningful contribution to the understanding of how selected herbal additives influence the physicochemical, microbiological, bioactive properties, and sensory characteristics of kombucha, serving as a foundation for further research on functional fermented beverages.

## 4. Conclusions

This study showed that the addition of herbal infusions—*Mentha spicata*, *Hibiscus sabdariffa*, and *Clitoria ternatea*—significantly affects the physicochemical, microbiological, and sensory properties of green tea-based kombucha drinks. Each of the additives used contributed unique characteristics to the final product, modifying the fermentation process, the profile of bioactive compounds, and the sensory profile as assessed by a panel of experts. *Hibiscus sabdariffa* significantly increased the content of polyphenols and flavonoids, enriched the flavor profile with sweet and fruity notes, and showed potential antimicrobial activity. *Clitoria ternatea* achieved the highest overall sensory quality and the highest number of lactic acid bacteria, which may suggest its probiotic potential. *Mentha spicata* imparted a characteristic mint aroma and the lowest caffeine content, which may be beneficial for people who prefer beverages with a milder stimulating effect. All variants were microbiologically safe, and it can be suggested that the herbal additives exhibited have a mild antimicrobial activity, likely associated with the presence of polyphenols and flavonoids. Sensory tests, conducted using the QDA method and an electronic tongue, confirmed significant differences in the flavor profiles and sensory quality between the variants, indicating the influence of herbal additives on the perceptual characteristics of the beverage. The results obtained represent a significant contribution to the development of knowledge on kombucha fermentation using plant raw materials and confirm that appropriately selected additives can significantly enrich the nutritional and sensory value of the beverage. Further research is needed, particularly in the area of biological replicates, extended fermentation times, functional tests confirming antioxidant and antimicrobial activity, and integrated multidimensional analyses to better understand the relationships between the chemical and microbiological composition and the sensory properties of the product. In summary, the results obtained confirm that enriching kombucha with selected herbal additives can be an effective tool in designing beverages with increased functional value and a diverse sensory profile, which opens up new perspectives for further research in this area.

## Figures and Tables

**Table 1:** pH values of the tested kombucha samples on individual days of fermentation.

Day	pH			
	K1	K2	K3	K4
0	4.15 ± 0.01 ^b^	4.40 ± 0.00 ^a^	3.47 ± 0.02 ^d^	3.99 ± 0.04 ^c^
3	3.27 ± 0.01 ^c^	3.64 ± 0.01 ^a^	3.23 ± 0.02 ^c^	3.32 ± 0.01 ^b^
4	3.22 ± 0.02 ^b^	3.40 ± 0.01 ^a^	3.17 ± 0.01 ^c^	3.19 ± 0.01 ^b^
5	3.21 ± 0.01 ^b^	3.40 ± 0.01 ^a^	3.19 ± 0.02 ^b^	3.19 ± 0.01 ^b^

^a, b, c, d^—mean values in rows marked with different letters differ significantly (*p* ≤ 0.05). K1—control, K2—30% mint, K3—30% hibiscus, K4—30% *clitoria*.

**Table 2:** Extract content in the tested kombucha samples.

Sample	Extract Content [%]
K1	6.50 ± 0.16 ^c^
K2	7.00 ± 0.22 ^b^
K3	7.83 ± 0.05 ^a^
K4	7.83 ± 0.05 ^a^

^a, b, c^—mean values in rows marked with different letters differ significantly (*p* ≤ 0.05). K1—control, K2—30% mint, K3—30% hibiscus, K4—30% *clitoria*.

**Table 3:** Bioactive compounds and their amounts in the tested samples.

Bioactive Compound [mg/L]	K1	K2	K3	K4
Total polyphenols	171.57 ± 2.72 ^b^	121.10 ± 4.07 ^c^	291.00 ± 3.95 ^a^	157.17 ± 3.07 ^b^
Total phenolic acids	84.92 ± 1.09 ^b^	63.09 ± 1.86 ^c^	82.87 ± 2.73 ^a^	92.35 ± 1.95 ^b^
Gallic acid	1.76 ± 0.10 ^a^	1.42 ± 0.10 ^b^	1.75 ± 0.01 ^a^	1.19 ± 0.05 ^c^
Chlorogenic acid	17.92 ± 0.35 ^a^	10.61 ± 0.25 ^b^	9.60 ± 1.99 ^b^	16.33 ± 0.19 ^a^
Caffeic acid	39.81 ± 0.44 ^a^	17.57 ± 0.44 ^c^	27.15 ± 0.35 ^b^	29.26 ± 0.72 ^b^
p-coumaric acid	3.05 ± 0.07 ^c^	19.04 ± 0.66 ^b^	28.95 ± 0.35 ^a^	29.90 ± 0.90 ^a^
Ferulic acid	11.31 ± 0.21 ^a^	6.57 ± 0.26 ^c^	8.23 ± 0.18 ^b^	7.37 ± 0.13 ^b^
Vanillic acid	8.90 ± 0.05 ^a^	4.04 ± 0.10 ^b^	5.64 ± 0.05 ^c^	5.71 ± 0.04 ^c^
t-cinnamic acid	2.17 ± 0.13 ^b^	3.85 ± 0.23 ^a^	1.55 ± 0.08 ^c^	2.58 ± 0.15 ^b^
Total flavonoids	86.65 ± 2.04 ^b^	58.01 ± 2.36 ^c^	208.13 ± 1.54 ^a^	64.83 ± 1.31 ^c^
Epigallocatechin	1.41 ± 0.20 ^a^	0.94 ± 0.05 ^b^	1.49 ± 0.02 ^a^	1.62 ± 0.11 ^a^
Catechin	24.18 ± 1.24 ^b^	11.77 ± 0.47 ^c^	165.82 ± 0.51 ^a^	19.44 ± 1.20 ^b^
Quercetin-3-O-rutinoside	24.08 ± 0.30 ^a^	12.59 ± 0.26 ^b^	15.40 ± 0.16 ^b^	20.00 ± 0.32 ^a^
Naringin	3.33 ± 0.09 ^b^	1.91 ± 0.13 ^c^	2.29 ± 0.09 ^c^	4.13 ± 0.04 ^a^
Kaempferol-3-O-glucoside	28.98 ± 0.37	15.03 ± 0.60 ^c^	18.48 ± 0.40 ^b^	15.48 ± 0.19 ^c^
Quercetin	2.34 ± 0.15 ^b^	11.16 ± 0.75 ^a^	1.92 ± 0.13 ^c^	2.33 ± 0.12 ^b^
Kaempferol	2.33 ± 0.01 ^b^	4.60 ± 0.43 ^a^	2.72 ± 0.32 ^b^	1.82 ± 0.03 ^c^
Caffeine	321.91 ± 3.50 ^a^	114.34 ± 5.47 ^c^	162.60 ± 1.02 ^b^	172.08 ± 1.18 ^b^

^a, b, c^—mean values in rows marked with different letters differ significantly (*p* ≤ 0.05). K1—control, K2—30% mint, K3—30% hibiscus, K4—30% *clitoria*.

**Table 4:** Results of microbiological analysis of kombucha beverages [log CFU/mL].

Kombucha Beverages	TVC	AAB	LAB	Yeast and Mould	ENT	BC
K1	7.08 ± 0.09 ^a^	3.03 ± 0.05 ^a^	<1.00	6.17 ± 0.04 ^a^	<1.00	<1.00
K2	7.04 ± 0.05 ^a^	2.26 ± 0.41 ^b^	<1.00	6.09 ± 0.08 ^a^	<1.00	<1.00
K3	6.38 ± 0.21 ^b^	2.06 ± 0.10 ^b^	2.24 ± 1.97 ^a^	6.11 ± 0.03 ^a^	<1.00	<1.00
K4	6.35 ± 0.17 ^b^	2.39 ± 0.46 ^b^	3.61 ± 3.16 ^b^	6.32 ± 0.22 ^a^	<1.00	<1.00

Explementary notes: <1.00—counts below the detection limit of the plating method; ^a, b^—mean values in rows marked with different letters differ significantly (*p* ≤ 0.05). K1—control, K2—30% mint, K33—0% hibiscus, K4—30% *clitoria*.

**Table 5:** Sensory profiling of kombucha samples (n = 20).

Attributes	Sample Code			
	K1	K2	K3	K4
Visual attribute				
clarity	4.74 ^b^	7.60 ^a^	7.78 ^a^	7.11 ^a^
Odor attributes				
acetic	4.87 ^a^	3.09 ^b^	4.46 ^a^	4.34 ^a^
sharp	3.76	3.10	2.94	3.20
sour	3.49	2.90	3.30	3.03
fermentative	4.22 ^a^	2.41 ^b^	3.51 ^a^	3.75 ^a^
sweet	2.50	2.85	2.95	2.61
fruit	2.67	2.53	3.30	3.25
tea	2.18	1.98	2.01	1.97
mint	0.41 ^b^	7.54 ^a^	0.13 ^b^	0.26 ^b^
herbal	2.58 ^ab^	3.22 ^a^	2.55 ^ab^	2.42 ^b^
Taste/flavor attributes				
acetic	5.93 ^a^	5.09 ^b^	5.23 ^b^	4.33 ^c^
sharp	4.91 ^a^	4.83 ^a^	4.17 ^b^	3.24 ^c^
tingling	4.49 ^a^	3.79 ^b^	4.20 ^ab^	2.41 ^c^
sour	5.31 ^a^	4.98 ^a^	4.49 ^b^	3.88 ^c^
sweet	2.63 ^c^	3.35 ^b^	4.70 ^a^	4.02 ^ab^
fruit	2.91 ^b^	2.89 ^b^	4.13 ^a^	4.35 ^a^
tea	2.41	2.08	2.07	2.32
mint	0.27 ^b^	7.35 ^a^	0.20 ^b^	0.27 ^b^
herbal	1.95	2.46	1.88	2.06
pungent	3.69 ^a^	3.03 ^ab^	2.83 ^b^	2.68 ^b^
overall sensory quality	5.08 ^d^	6.73 ^b^	6.24 ^c^	7.37 ^a^

^a, b, c, d^—mean values in rows marked with different letters differ significantly (*p* ≤ 0.05). K1—control, K2—30% mint, K3—30% hibiscus, K4—30% *clitoria*.

## Data Availability

All data supporting the findings of this study are included within the article. Additional raw data or processed datasets are available from the corresponding author upon reasonable request.
